# Wisdom of patients: predicting the quality of care using aggregated patient feedback

**DOI:** 10.1136/bmjqs-2017-006847

**Published:** 2017-09-28

**Authors:** Alex Griffiths, Meghan P Leaver

**Affiliations:** 1 Centre for Analysis of Risk and Regulation, London School of Economics and Political Science, London, UK; 2 Department of Psychological and Behavioural Science, London School of Economics and Political Science, London, UK

**Keywords:** patient-centred care, patient satisfaction, quality measurement, risk management

## Abstract

**Background:**

The Care Quality Commission (CQC) is responsible for ensuring the quality of healthcare in England. To that end, CQC has developed statistical surveillance tools that periodically aggregate large numbers of quantitative performance measures to identify risks to the quality of care and prioritise its limited inspection resource. These tools have, however, failed to successfully identify poor-quality providers. Facing continued budget cuts, CQC is now further reliant on an ‘intelligence-driven’, risk-based approach to prioritising inspections and a new effective tool is required.

**Objective:**

To determine whether the near real-time, automated collection and aggregation of multiple sources of patient feedback can provide a collective judgement that effectively identifies risks to the quality of care, and hence can be used to help prioritise inspections.

**Methods:**

Our *Patient Voice Tracking System* combines patient feedback from NHS Choices, Patient Opinion, Facebook and Twitter to form a near real-time collective judgement score for acute hospitals and trusts on any given date. The predictive ability of the collective judgement score is evaluated through a logistic regression analysis of the relationship between the collective judgement score on the start date of 456 hospital and trust-level inspections, and the subsequent inspection outcomes.

**Results:**

Aggregating patient feedback increases the volume and diversity of patient-centred insights into the quality of care. There is a positive association between the resulting collective judgement score and subsequent inspection outcomes (OR for being rated ‘Inadequate’ compared with ‘Requires improvement’ 0.35 (95% CI 0.16 to 0.76), Requires improvement/Good OR 0.23 (95% CI 0.12 to 0.44), and Good/Outstanding OR 0.13 (95% CI 0.02 to 0.84), with p<0.05 for all).

**Conclusions:**

The collective judgement score can successfully identify a high-risk group of organisations for inspection, is available in near real time and is available at a more granular level than the majority of existing data sets. The collective judgement score could therefore be used to help prioritise inspections.

## Introduction

The Care Quality Commission (CQC) is the independent regulator responsible for ensuring the quality of over 30 000 health and social care providers in England.^1^ This includes 150 acute and specialist trust—administrative groupings of one or more hospitals in a geographical region—that provide the majority of hospital-based care for the National Health Service (NHS).^2^ Rather than adopt a random or cyclical approach to prioritising its limited inspection resource, CQC is legally required to operate an intelligence-led, risk-based approach.^3^ With its annual budget to be cut by £32 million (13%) by 2019,^4^ CQC has stated it plans to further rely on data to ‘target our resources where the risk to the quality of care provided is greatest’.^5^

To support its risk-based approach, CQC has relied on a succession of statistical surveillance tools aggregating large volumes of trust-level performance data. Based on the Healthcare Commission’s surveillance tool, ‘Quality and Risk Profiles’ (QRPs) aggregated hundreds of weighted, z-scored quantitative indicators and manually-coded qualitative comments, each mapped to one of CQC’s 16 ‘essential standards’ of care.^6^ QRPs automatically generated a risk score for each of the 16 ‘essential standards’ and were updated simultaneously for all trusts nine times a year. Although comprehensive, QRPs faced heavy criticism for the quality and timeliness of information they contained, their complexity and their inability to achieve their core function—to effectively predict risks to the quality of care.^7 8^

Following the critical Francis Inquiry into the high-profile failings at Mid-Staffordshire Trust, CQC overhauled its regulatory approach including its statistical surveillance tool.^9^ QRPs were replaced by the far simpler ‘Intelligent Monitoring’ (IM) tool. IM generated a single trust-level ‘risk score’ based on approximately 150 equally weighted performance indicators that were simultaneously updated for all trusts every 5 months.^10^ Two sources of patient feedback data were included in the final version of IM, the proportion of ‘Share Your Experience’ comments submitted via CQC’s website that were manually coded as ‘negative’,^11^ and standardised scores from select questions on the annual inpatient survey. IM was unable to successfully identify high-risk trusts with its predictions proving wrong more often than not.^12^ Like QRPs, concerns were expressed over the quality, timeliness and granularity of information IM contained.^13–15^ CQC has now begun developing its ‘CQC Insight’ surveillance tool to replace IM.^15^

One widely supported solution to identifying risks to the quality of care is to make use of abundant patient feedback in the NHS.^8 16–20^ In addition to providing a different perspective on quality to traditional metrics, such as readmission rates and staffing levels,^21 22^ patient feedback from online rating pages NHS Choices and Patient Opinion, and via Twitter and Facebook, is available in a more timely manner (near real time), and at a more granular (hospital) level, than the data typically used by CQC. Patient feedback therefore has the potential to help address the perennial concerns over the timeliness and granularity of data used to identify risks to the quality of care.

Timely and granular information, however, will be of little use if it does not reflect the quality of care. Existing research into the use of patient feedback to identify quality concerns has compared individual sources with quality measures at a fixed point in time and has shown encouraging, if mixed, results. In the UK, an association has been demonstrated between NHS Choices ratings, Inpatient Survey scores and select clinical outcome measures.^23^ No association has been found between the sentiment of tweets mentioning NHS trusts and mortality rates or NHS Inpatient Survey scores; however, the study analysed the sentiment of all tweets whether they related to the quality of care or not.^24^ In the USA, an association was found between Yelp ratings, the Hospital Consumer Assessment of Healthcare Providers and Systems (HCAHPS) survey, and to a lesser extent with select clinical outcomes.^25^ Furthermore, an association between standardised Facebook ratings and readmission rates was identified.^26^ A weak association was found between 30-day readmission rates and the sentiment of care quality-related tweets, although no association was found between sentiment and the HCAHPS survey.^27^

Individual sources of patient feedback being biased towards certain demographics were identified as a barrier to the more effective measurement of quality in each of these studies. One possible way to overcome the bias of individual sources of patient feedback is to combine them. In the UK, Twitter has 15.8 million active users, 7.9 million (50%) of whom are aged below 35, predominantly of higher socioeconomic status. Facebook has 37.5 million active users, 21 million (56%) of whom are aged 35 or over, with users of a lower socioeconomic status over-represented.^28 29^ Demographics data are not available for NHS Choices; however, the wider site, which includes health information, received 583 million unique visits in 2015, nearly 10 visits for every member of the UK.^30^ Combining these high-volume, disparate sources of patient feedback in near real time is technically challenging however, and has yet to be successfully operationalised.

The aim of our study is to determine whether the near real-time, automated collection and aggregation of patient feedback can provide a collective judgement that effectively identifies risks to the quality of care, and hence can be used to help prioritise CQC inspections. This study furthers the existing research in a number of ways. Unlike previous research, this combines multiple sources of patient feedback, it looks at the more granular, hospital-level feedback, and it measures the association between patient feedback and other quality measures at hundreds of points over more than 3 years, rather than a fixed point in time. Moreover, the patient feedback used is contemporary having occurred within 90 days of the start of the inspection, and covers a greater volume and diversity of trusts and hospitals as a result of increased engagement with social media by the NHS.

## Methods

Our analysis measures the statistical relationship between a time-limited collective judgement score (CJS) formed of patient feedback from multiple sources at the start of comprehensive CQC inspections, and the subsequent outcome of those inspections. The data sets and methods for this analysis are detailed below.

### Dependent variable

In October 2013, CQC introduced a new comprehensive inspection regime and by March 2017 had visited all NHS hospitals and trusts in England.^31^ Under that regime, inspections are conducted by large teams of specialist inspectors, clinicians and ‘experts by experience’ who assess individual hospital services against five ‘key questions’: is the service ‘safe’, ‘effective’, ‘caring’, ‘responsive to people’s needs’ and ‘well led’?^32^ Based on their inspection teams’ on-site visits, CQC then awards one of four possible ordinally ranked ratings for each core service within a hospital:

1. Outstanding
2. Good
3. Requires improvement
4. Inadequate

CQC then aggregates those service-level ratings using an unpublished, rules-based approach to assign hospital-level ratings, which, in turn, are further aggregated, using a similar rules-based approach, to generate an overall trust-level rating.

Up to 12 March 2017, CQC published 204 and 339 trust and hospital-level ratings, respectively. The majority of trusts and hospitals have been rated only once under the new inspection regime. Those inspected more than once were typically, although not always, rated poorly on their first inspection and have since been inspected again to check for improvements. A minority of inspections contained no overall rating. This occurred when CQC deemed there was insufficient evidence to produce a rating, or where the inspection team focused on a specific aspect of care and therefore could not judge the overall quality of care.

### Independent variables

We obtained details of all NHS acute and specialist trusts and their hospitals, including the URL for their patient feedback submitted to NHS Choices, from the NHS Choices application programming interface (API) in February 2016. We then searched Google for each organisation’s Twitter and Facebook details. Where no page was found, the organisation’s own website was checked. The details for each data source and how they were combined are described below and in table 1.

**Table 1:** Summary of the three sources of patient feedback used to form the collective judgement score

	NHS Choices	Facebook	Twitter
Time period data available	1 January 2013 to 12 March 2017	1 January 2013 to 12 March 2017	21 February 2016 to 12 March 2017
Total number of comments collected	76 493	69 427	1 303 085
Unique comments suitable for study	76 493	69 427	20 914
Unique comments suitable for study covering 1 March 2016 to 28 February 2017	20 270	19 572	19 771
CQC-rated hospitals with an account/page	245	204	13
CQC-rated trusts with an account/page	148	132	142
Mean sentiment score (from 1 to 5)	3.85	4.13	4.28

CQC, Care Quality Commission.

#### NHS Choices (including Patient Opinion)

NHS Choices is a government-run website that captures unsolicited feedback for all NHS trusts and hospitals. Users can leave free-text feedback and rate how likely they are to recommend the organisation to friends or family in need of similar care on a scale from 1 (extremely unlikely) to 5 (extremely likely) stars. Patient Opinion is a social enterprise which provides a similar function to NHS Choices including the ability to leave qualitative feedback and recommend the service on the same scale. Feedback posted to Patient Opinion is shared with, and appears on, the NHS Choices website. Comments left on either site are actively moderated before being displayed with references to individuals and speculation removed.^33–35^ All patient feedback posted between 1 January 2013 and 12 March 2017 inclusive was sourced via NHS Choices’ API.

#### Facebook

Facebook directly captures patient feedback in two ways. First, organisations can create official Facebook pages with a ‘Reviews’ section. Anyone accessing the page can leave a review, which must contain a rating from 1 to 5 stars with additional free-text feedback being optional. Second, organisations that users frequently search for automatically have an unofficial page generated that includes a similar review function to official pages. Comments for both page types are reactively moderated; once posted, comments cannot be amended and will only be removed if they breach Facebook’s Community Standards.^36^ We sourced all reviews posted from 1 January 2013 to 12 March 2017 using a custom Python script.

#### Twitter

Starting in February 2016, we queried the Twitter API at regular intervals of no more than 7 days to ensure complete coverage from the time and volume-limited data source. Unlike NHS Choices and Facebook data, not all comments related to the quality of care. To ensure only original feedback was included, and that the results were not biased by the popularity of users, all retweets and tweets generated by trusts or hospitals were removed.

After 6 months of data had been collected, a representative pilot data set of 1000 tweets was jointly coded by AG and MPL and the five classifications of tweet detailed in table 2 were agreed. Tweets that were clearly not original feedback from patients or their friends and family were classed as ‘Not patient feedback concerning the quality of care’ to prevent staff and promotional activities biasing the data set. A second representative sample comprising 10% (23 014) of all tweets, not retweeted or generated by the organisation in question, was then coded by AG, with just over 25% (5869) of AG’s coding second-coded by MPL to check reliability. Inter-rater reliability was ‘substantial’ (κ=0.6–0.8) for all classifications except for the rarest, ‘safety’, where it was ‘moderate’ (κ=0.4–0.6).^37^ All differently coded tweets were then jointly reviewed and complete agreement was reached.

**Table 2:** The count and reliability of each category of tweet coded by both AG and MPL

Tweet classification		N	Inter-rater reliability (Cohen’s κ)	p Value
Patient feedback concerning the quality of care	Patient experience and effectiveness	348	0.64	<0.001
	Environment and facilities	67	0.67	<0.001
	Timeliness and access	90	0.75	<0.001
	Safety	13	0.45	<0.001
Not patient feedback concerning the quality of care		5351	0.67	<0.001
Total		5869		

The next stage was to automate the classification of the tweets. We first oversampled the data to account for the class imbalance in care-quality related tweets^38 39^ and set aside 25% of the data set for model testing. We then developed a variety of models using fivefold cross-validation and a tuning grid of model-specific parameters to classify the tweets. The chosen model achieved overall precision, recall and F_1_ scores of 0.97.^40^

Tweets that were not classed as patient feedback concerning the quality of care were then excluded from the study and the sentiment of the remaining tweets was scored using the same 1 to 5-star scale as the NHS Choices and Facebook data. Initial attempts to use standard sentiment dictionaries such as SentiWordNet proved ineffective, likely due to the healthcare-specific lexicon and non-prose style of tweets. Instead, the same modelling approach used for classifying the tweets was used to identify sentiment. The chosen model predicted the 1 to 5-star rating of scored comments with 79% accuracy, rising to 91% accuracy when estimating the sentiment to within 1 star, and was used to assign a star rating to all care-quality related tweets. Further details of the modelling are available in the online supplementary technical appendix.

10.1136/bmjqs-2017-006847.supp1 Supplementary file 1

### Combining and analysing the data

With a date and score ranging from 1 (lowest) to 5 (highest) stars associated with every item of patient feedback, a CJS could be calculated for all trusts and hospitals on any given date where sufficient patient feedback was available. A 90-day moving average was chosen as a balance between the CJS reflecting the more recent patient feedback, and being too volatile and overly influenced by a small number of recent items of patient feedback leading to unnecessary inspections being triggered. The 90-day CJS on the start date of each trust and hospital-level inspection was then paired with the overall rating from the inspection. Of the 543 trusts and locations rated by CQC, 87 were then removed from the data set as their 90-day CJS comprised fewer than 10 items of patient feedback. The majority of organisations removed were specialist units within a trust.

As trust-level ratings are an aggregation of hospital-level ratings they are not independent, and combining them for a single model would violate the assumptions of the regression model. The relationships between the 90-day CJS at the start of an inspection and the subsequent trust and hospital-level inspection ratings were therefore assessed using two distinct ordinal logistic regression models both of which used random intercepts and random coefficients.

## Results

**Figure 1:**
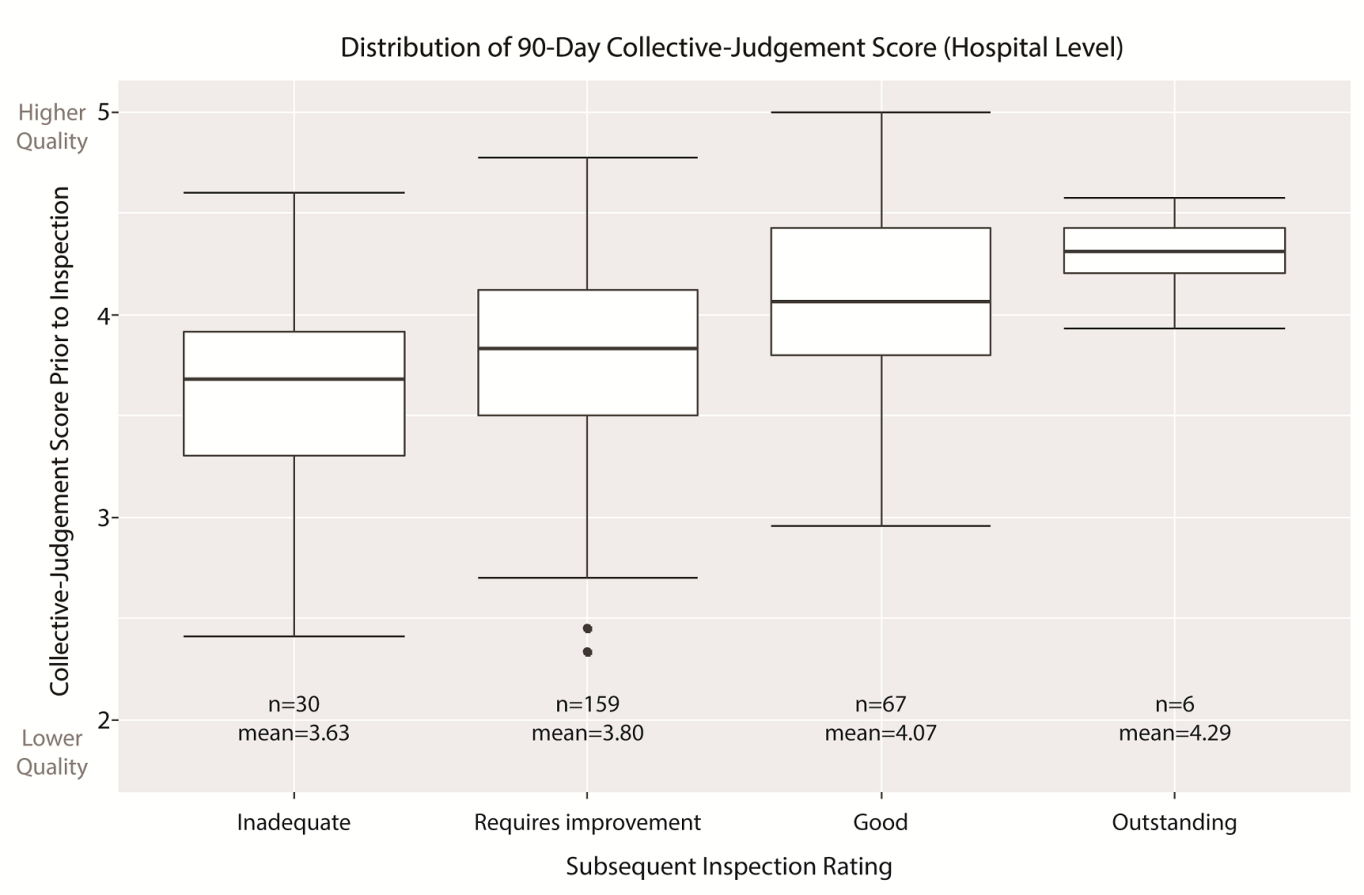
Box plot of collective judgement scores grouped by inspection rating.

Figure 1 shows the distribution of the 90-day CJS on the first day of hospital-level inspections grouped by the subsequent overall rating. For each improved inspection rating, the mean and median CJS increases, indicating that on average patient feedback is better for hospitals the CQC subsequently awards a higher rating. The better the patient feedback in the 90 days prior to a CQC inspection, the greater the likelihood of a more positive overall rating.

The ordinal (cumulative) regression model detailed in table 3 describes the probability of a hospital being awarded a prescribed CQC rating given a particular CJS score and confirms the statistical significance of the association between increasing CJS and improved inspection ratings. The same significant relationship identified for hospitals is also present for trusts when considered in isolation. Further details about the regression model can be found in the online supplementary technical appendix.

**Table 3:** The regression coefficients and associated SEs, ORs and associated 95% CIs and p values for the hospital-level ordinal (cumulative) logistic regression model

	Beta (SE)	95% CI for OR			Pr(>|z|)
		2.5%	OR	97.5%	
Inadequate (intercept)	1.89 (1.47)				0.199
Requires improvement (intercept)	6.74 (1.31)				0.000
Good (intercept)	12.00 (4.05)				0.003
Inadequate (CJS)	−1.04 (0.40)	0.16	0.35	0.77	0.008
Requires improvement (CJS)	−1.47 (0.33)	0.12	0.23	0.44	0.000
Good (CJS)	−2.02 (0.94)	0.02	0.13	0.84	0.032

CJS, collective judgement score.

The overlapping CJS across all four rating categories show that, despite the tendency for hospitals awarded a higher rating to have a higher 90-day CJS, it is not a perfect predictor of the outcome of inspections. A small number of ‘Inadequate’ hospitals have a high CJS, as do a greater number of ‘Requires improvement’ hospitals. Conversely, all ‘Outstanding’ hospitals have an above average CJS, suggesting patients are better collective judges of organisations performing well than they are of organisations performing poorly.

As of 12 March 2017, CQC had published 55 trust-level and 88 hospital-level inspection reports that contained an overall rating and where the inspection began in 2016. With CQC adopting a more targeted approach, and facing budget cuts, the number of inspections it conducts is likely to fall. Were CQC to prioritise the 50 trusts or 80 hospitals with the lowest CJS in the data set the outcome would be as described in table 4.

**Table 4:** A contingency table showing the number of hospitals and trusts that would have been inspected, and the outcome of those inspections, had the 50 trusts or 80 hospitals with the lowest 90-day collective judgement score been inspected

		Inadequate	Requires improvement	Good	Outstanding	Total
Hospital level	Higher priority (inspect)	14	56	10	0	80
	Lower priority (cannot inspect)	16	103	57	6	182
	Total	30	159	67	6	262
Trust level	Higher priority (inspect)	9	34	7	0	50
	Lower priority (cannot inspect)	13	83	41	9	146
	Total	22	117	48	9	196

For hospitals, 70 out of the 80 inspections would result in a rating of ‘Inadequate’ or ‘Requires improvement’. Moreover, only 10 of the 67 ‘Good’ hospitals and none of the 6 ‘Outstanding’ hospitals would be unnecessarily burdened with inspection. Similarly, 43 out of the 50 inspections of trusts would result in a rating of ‘Inadequate’ or ‘Requires improvement’ and only 7 of the 48 ‘Good’ trusts and none of the 9 ‘Outstanding’ trusts would be unnecessarily burdened with inspection. This represents a precision rate (the proportion of high-risk organisations that were subsequently rated as ‘Inadequate’ or ‘Requires improvement’) of 87.5% and 86% for hospitals and trusts, respectively. The issue for CQC is that, even with a tool that was 100% precise, the constrained number of inspections would still mean that 109 out of the 189 hospitals that were rated ‘Requires improvement’ or ‘Inadequate’ would not be inspected. In the unlikely event CQC’s capacity for inspection were to double, the CJS would remain an effective tool with precision rates of 81% for both hospital and trust-level inspections. This is a meaningful improvement on the 72% and 71% that would be achieved at hospital and trust levels, respectively, by chance alone.

## Discussion

### Statement of principal findings

The study establishes that the near real-time, automated collection and aggregation of patient experiences from multiple sources, including social media, can provide a collective judgement that effectively identifies a high-risk group of organisations, and hence can be used to help prioritise inspections.

### Policy implications

No combination of indicators, quantitative or qualitative, will ever perfectly predict the outcome of inspections. If they could, there would be little need for expensive inspections. Yet CQC does not have the resource to inspect each hospital every year. It therefore prioritises inspections as best it can with the data that are available. The CJS can support this data-driven approach in three ways.

First, while the collated patient feedback cannot perfectly identify poorly performing organisations, it can identify those organisations that are *most likely* to be performing poorly. Indeed, not a single organisation with a CJS below 2.95 was rated ‘Good’ or ‘Outstanding’. Moreover, 89 out of 130 (68%) ‘Good’ or ‘Outstanding’ organisations had an above average CJS at the start of their inspection, compared with 147 out of 328 (44%) ‘Requires improvement’ or ‘Inadequate’ organisations. Second, much of the patient feedback is available at hospital level and can therefore be used to support more focused inspections than the trust-level data currently used.

**Figure 2:**
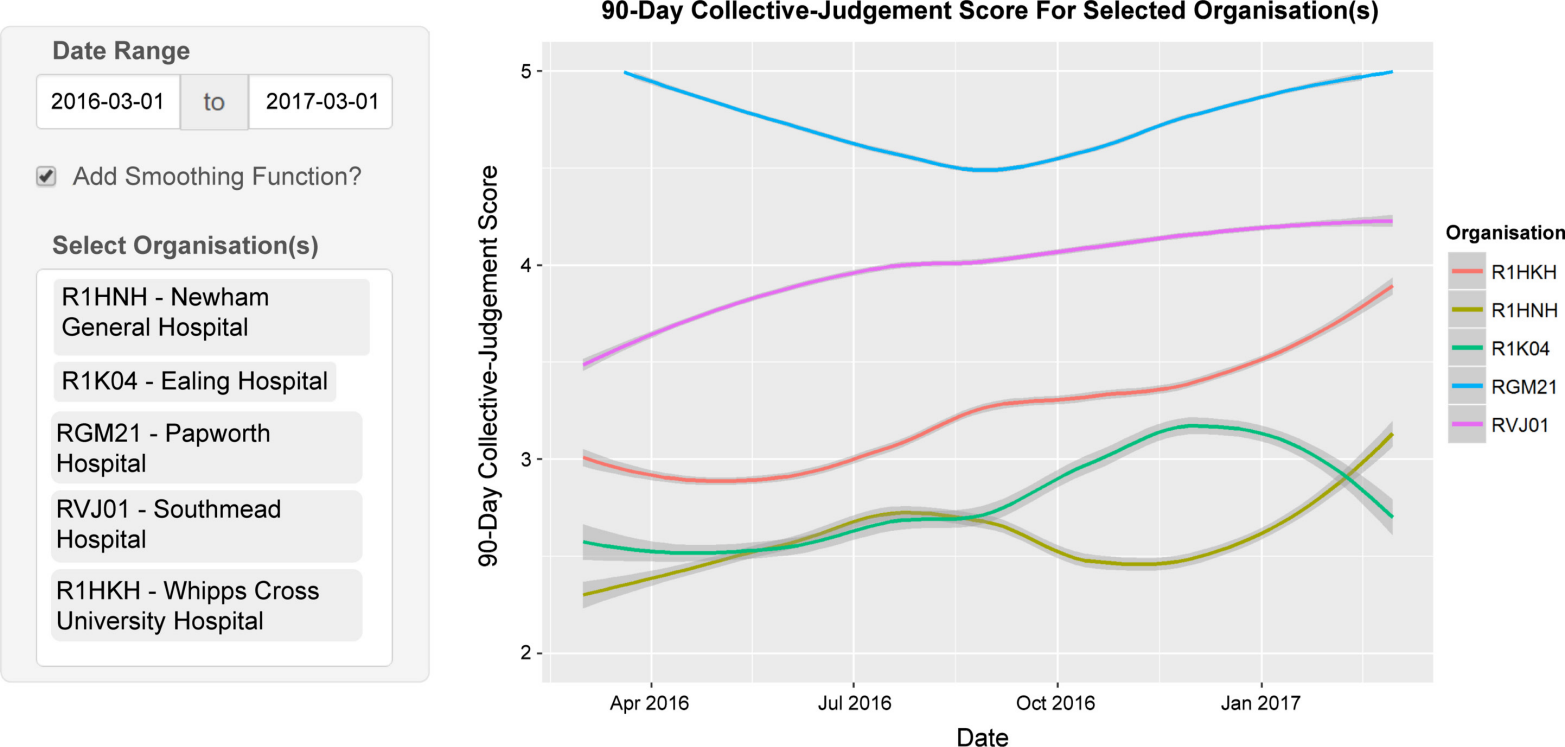
The Loess-smoothed 90-day collective judgement score for six NHS (National Health Service) organisations over a 1-year period taken from the Patient Voice Tracker System developed by the authors.

Third, unlike CQC’s statistical surveillance tools that comprised quarterly or annual performance measures that were slow to gather and process, and updated at most nine times a year, the CJS can be updated in near real time with up-to-date patient feedback. One advantage of the more timely data is the ability to spot rapid changes, such as the extreme examples of Ealing Hospital (R1K04) and Newham General Hospital (R1HNH) illustrated in figure 2. While some patients must experience poor care for the CJS to prompt action, the number may be far fewer than when care is monitored solely by large quarterly data collections.

Not only does the more timely data have the potential to benefit regulators and by extension patients, whose experience make-up the signals they eventually act upon, hospitals benefit too. Healthcare settings are complex and unpredictable and the continuous identification of high-quality care (which is more common than low-quality care) can allow organisations to leverage information on good practice for mitigating, rather than reducing, poor-quality care. Such information enables organisations to proactively improve their resilience, and is consistent with a ‘Safety II’ approach to maintaining a high quality of care.^41^

Irrespective of these stated benefits, it would be unwise to rely solely on patient feedback to prioritise inspections. Doing so would disregard large volumes of potentially valuable quality information including mortality and readmission rates, waiting times and staffing levels. Although CQC’s IM tool has not been successful, other means of aggregating quantitative data have shown promise in the past^42^ and it is probable that such data considered in conjunction with patient feedback provide the best means to effectively identify risks to the quality of care and prompt further investigation.^17^

The results of this study raise two questions about patients’ collective judgement which require further consideration. First, why is it that, collectively, patients are better able to identify organisations that CQC will rate well, than organisations they will rate poorly? One possibility is that the issues CQC found in hospitals and trusts they rated poorly but had a high CJS were less patient-facing, or impacted patients less directly, than others. An alternative explanation is that CQC ratings, and in particular the negative ratings, are unreliable. Concerns over the reliability of CQC’s inspection outcomes are not new,^43^ but the comprehensive inspections comprise large teams of specialist inspectors, clinicians and ‘experts by experience’, have received significant support from the sector.^9 14 44^

The second question is how are patients, the vast majority of whom will have no clinical training, able to predict the outcome of comprehensive quality inspections? There are several possible non-exclusive explanations. A growing volume of research suggests that, in addition to the more service-orientated aspects of care such as dignity and respect, cleanliness and timeliness, patients are able to accurately assess some of the more clinical aspects of their care.^45–47^ This insight may be enhanced by the ‘wisdom of crowds’ phenomenon which states that, under the right circumstances, groups can be remarkably insightful, even if the majority of people within a group are not especially well informed.^48^ While individuals seldom have all the necessary facts to make an accurate assessment, and are subject to numerous heuristics and biases, when their assessments are aggregated in the right way, the collective assessment is often highly accurate. Combining multiple sources increases the likelihood that patients’ collective judgement will be accurate as it increases both the volume and diversity of feedback. It may also be the case that aspects of care that are more easily assessed reflect accurately on those that are not. The poor management which results in impolite staff and poor timekeeping may also impact clinical effectiveness. Finally, another possibility is that CQC inspectors focus on the satisfaction of patients at the expense of clinical measures that patients may be less able to accurately assess for themselves.

Regardless of the reason for the agreement between the CJS and CQC inspection ratings, it is probable that its adoption will result in some unwelcome behaviour. First, some degree of ‘gaming’ is inevitable.^49^ While this cannot be eliminated entirely, the effects can be minimised: meta-data is available with all feedback and repeated comments from certain users, or multiple supposed patients at similar times or using similar language can be easily detected. Moreover, the sheer volume of comments means attempts by individuals to manually game the data will have minimal impact. Second, both organisations and patients may change their behaviour knowing the potential consequences of feedback. Some changes in behaviour, such as more courteous staff and better communicated appointments, may be positive; but there may also be a pressure to provide unnecessary or ineffective treatments to placate newly empowered patients. With the collection of patient feedback well established in the NHS, the effect on patients and organisations of formally collating additional feedback may be limited.

### Strengths and weaknesses of the study

Our study has a number of strengths. First, it combines multiple sources of patient feedback to increase the volume and diversity of patient feedback. Second, it accurately classifies tweets excluding those that are not patient feedback related to the quality of care. Third, the inspection ratings used as an independent variable are comprehensive and reflect the quality of care throughout at multiple points over more than 3 years, rather than a snapshot provided by other quality measures such as annual surveys. Fourth, it made use of the more granular hospital-level feedback and ratings, and therefore suffers less from the overaggregation of information concerning large, complex organisations.

Further to the potential challenges of gaming detailed above, our study has four constraints. First, Twitter data were only available from February 2016 onwards and so have only been included for inspections starting after this point; this is due to the time-limited nature of Twitter’s API. Second, the class imbalance in the data, with 61% (277) of all organisations in the study being rated as ‘Requires improvement’ and only 3% (14) being rated as ‘Outstanding’, makes drawing robust conclusions harder than would be the case were the ratings more evenly distributed. Third, feedback via social media may be influenced by current affairs. Fourth, while combining multiple sources of patient feedback has reduced representative bias, some patient groups have undoubtedly remained under-represented.

### Unanswered questions and future research

There is still a lot to be learnt about the use of aggregated patient feedback. It is possible that the predictive power of the CJS could be improved by weighting patient feedback by age or source, standardising for organisation type, shortening or lengthening the time period for the CJS, or adding additional data sources. Moreover, the quality of care is neither objective nor one dimensional.^50^ Future research should aim to identify what feedback is most strongly associated with specific dimensions of quality in order to better target interventions. Identifying how patient feedback can be best combined with quantitative data, such as mortality rates and waiting times, in order to identify risk should be a priority for CQC.

## Conclusion

Aggregating patient feedback increases the volume and diversity of patient-centred insights into the quality of care. The resulting collective judgement can successfully identify a high-risk group of organisations for inspection, is available in near real time and is available at a more granular level than the majority of existing data sets. The near real-time, automated collection and aggregation of multiple sources of patient feedback should be used to help prioritise inspections.
