# Supplementary material for: Wisdom of patients: predicting the quality of care using aggregated patient feedback
Source: BMJ Qual Saf. 2017 Sep 28;27(2):110–8. doi: 10.1136/bmjqs-2017-006847 (PMC5867435; doi:10.1136/bmjqs-2017-006847)
Supplement: Supplementary file 1 [file bmjqs-2017-006847supp001.docx]

**The Wisdom of Patients: Predicting the Quality of Care using Aggregated Patient Feedback: Technical Appendix**

This technical appendix provides additional information on the models used to classify and score the tweets, the ordinal logistic regression model used analyse the relationship between the collective-judgement score and the subsequent outcome of CQC inspections, and information on how to access the data sources used in the study.

**Classifying Tweets**

A strong class imbalance was clear in the data with over 90% not being patient feedback that is related to the quality of care. The first stage was therefore to oversample the care-quality related tweets to allow the model a realistic chance of classifying them. Once this had been done the data was randomly split into two data sets: 75% was used to train the various potential models and 25% was used to test those models.

The tweets were then vectorised using Sci-Kit Learn’s TfidfVectorizer with the same library’s GridSearchCV function being used to help identify the best model. The following model parameters were varied:

| Parameter | Variants |
| --- | --- |
| Model type | Multinomial Naïve Bayes (MNB),  Support Vector Classifier (SVC)  Logistic regression (LR)  Stochastic Gradient Descent Classifier (SGD) |
| Regularisation (e.g. ‘alpha’ for MNB) | 1000,100,10,1,0.1,0.01,0.001 |
| Ngram_range | (1,1), (1,2), (1,3), (1,4), (1,5), (2,3), (2,4), (2,5) |
| Min_df | 1, 2, 3, 4 |
| Use_idf (whether or not) | False, True |

Table 1: The different model parameters explored to develop the model for classifying tweets.

Where ‘min_df’ determines the minimum number of times a word must appear in the data set to be included in the model and ‘use_idf’ determines whether or not the model should utilise inverse-document-frequency re-weighting.

The selected model was a multinomial naïve Bayes model with alpha = 0.1 and a vectoriser using the below parameters:

| Parameter | Value |
| --- | --- |
| token_pattern | r"[a-zA-Z]{2,30}" |
| analyzer | 'word' |
| min_df | 1 |
| max_df | 0.50 |
| ngram_range | (1,5) |
| use_idf | True |
| norm | ‘l2’ |

Table 2: The parameters for the chosen classification model.

A Bayesian prior was not used as the data was oversampled which would have meant future predictions from the model would expect a higher rate of care-quality related tweets than is correct.

The chosen model achieved overall precision, recall and F_1_ scores of 0.97.

**Scoring Tweets on a 1-5 Star Scale**

Following the poor performance of standard sentiment analysis dictionaries a custom approach was used to score the tweets. All Facebook and NHS Choices comments were combined and split into training and testing sets comprising 75% and 25% of the data respectively. Various models were then trained using 5-fold cross-validation to predict their star rating based on the text. After some exploratory analysis we determined there was little difference in performance between the various models but the multinomial naïve Bayes classifier was far quicker when applied to the large dataset and so was the only model considered in greater depth. The below parameters were considered:

| Parameter | Variants |
| --- | --- |
| Model type | Multinomial Naïve Bayes (MNB) |
| Regularisation (e.g. ‘alpha’ for MNB) | 1000,100,10,1,0.1,0.01,0.001 |
| Ngram_range | (1,1), (1,2), (1,3), (1,4), (1,5), (1,6), (1,7), (1,8), (2,3), (2,4), (2,5), (2,6), (2,7), (2,8) |
| Min_df | 1, 2, 3, 4, 5 |
| Use_idf (whether or not) | False, True |

Table 3: The different model parameters explored to develop the model for scoring tweets.

The selected model was a multinomial naïve Bayes model with alpha = 0.1 and a vectoriser using the below parameters:

| Parameter | Value |
| --- | --- |
| token_pattern | r"[a-zA-Z]{1,30}" |
| analyzer | 'word' |
| min_df | 2 |
| max_df | 0.50 |
| ngram_range | (1,4) |
| use_idf | True |
| norm | ‘l2’ |

Table 2: The parameters for the chosen classification model.

Again, a Bayesian prior was not used this time due to there being no way of knowing that the distribution of star ratings for Facebook and NHS Choices would be the same as for Twitter.

The chosen model achieved overall precision, recall and F_1_ scores of 0.74, 0.78 and 0.76 respectively rising to 0.91, 0.91 and 0.91 when estimating the sentiment to within one star.

**Ordinal Logistic Regression Model**

An ordinal logistic regression model was chosen because the categorical dependent variable was ordered, and therefore to use a multinomial logistic would represent a loss of information. Random intercepts and random coefficients were specified as it is improbable that all CQC ratings are equally likely at the intercept point and that a unit increase in the collective-judgement score would have a uniform effect on the likelihood of the organisation being rated any of the four possible CQC rating categories.

The model predicts the likelihood of an organisation being rated a particular CQC rating or worse based on their collective-judgement score. The below calculations show how these probabilities, and subsequently the probability an organisation will receive a specific rating, are calculated when a hospital’s collective-judgement score is 3:

$$P\left( hospital rated "Inadequate" \right|CJS=3)=\frac{e^{(1.89+(3 \times-1.04)}}{1+e^{(1.89+(3 \times-1.04)}}=0.226$$

$$P\left( hospital rated "Req Imp" or worse \right|CJS=3)=\frac{e^{(6.74+(3 \times-1.47)}}{1+e^{(6.74+(3 \times-1.47)}}=0.911$$

$$P\left( hospital rated "Req Imp" \right|CJS=3)=\frac{e^{(6.74+(3 \times-1.47)}}{1+e^{(6.74+(3 \times-1.47)}}- \frac{e^{(1.89+(3 \times-1.04)}}{1+e^{(1.89+(3 \times-1.04)}}=0.682$$

$$P\left( hospital rated "Good" or worse \right|CJS=3)=\frac{e^{(12.00+(3 \times-2.02)}}{1+e^{(12.00+(3 \times-2.02)}}=0.997$$

$$P\left( hospital rated "Good" \right|CJS=3)=\frac{e^{(12.00+(3 \times-2.02)}}{1+e^{(12.00+(3 \times-2.02)}}- \frac{e^{(6.74+(3 \times-1.47)}}{1+e^{(6.74+(3 \times-1.47)}}=0.086$$

$$P\left( hospital rated "Outstanding" \right|CJS=3)=1-\frac{e^{(12.00+(3 \times-2.02)}}{1+e^{(12.00+(3 \times-2.02)}}=0.003$$

**Accessing Twitter, Facebook and NHS Choices Data**

*Twitter*

Twitter data can be freely accessed via the RESTful API using a number of coding languages, or via purpose built tools.

Details of how users can access the Twitter API can be found [here](https://dev.twitter.com/rest/public) with detailed examples of how to query the API using Python [here](https://github.com/ptwobrussell/Mining-the-Social-Web-2nd-Edition/blob/master/ipynb/Chapter%209%20-%20Twitter%20Cookbook.ipynb). A more straightforward option for non-technical users is to make use of various Twitter monitoring tools including the user-friendly [Chorus Analytics](http://chorusanalytics.co.uk/) tool available free to registered academics.

*Facebook*

Facebook provide their own free-to-use API with full details available [here](https://developers.facebook.com/docs/marketing-api/using-the-api). Alternatively, the data can be gathered manually from Facebook pages.

*NHS Choices*

NHS Choices offer free access to all patient reviews via their API. Full information on how to register and use the API can be found [here](http://www.nhs.uk/aboutNHSChoices/professionals/syndication/Pages/Webservices.aspx).
